# Predictive Value of NT-proBNP, FGF21, Galectin-3 and Copeptin in Advanced Heart Failure in Patients with Preserved and Mildly Reduced Ejection Fraction and Type 2 Diabetes Mellitus

**DOI:** 10.3390/medicina60111841

**Published:** 2024-11-08

**Authors:** Raluca Diana Ianos, Mihaela Iancu, Calin Pop, Roxana Liana Lucaciu, Adriana Corina Hangan, Rodica Rahaian, Angela Cozma, Vasile Negrean, Delia Mercea, Lucia Maria Procopciuc

**Affiliations:** 1 Department of Cardiology, “Iuliu Hatieganu” University of Medicine and Pharmacy, 400001 Cluj-Napoca, Romania; ralu_yannosh@yahoo.com; 2 Medical Informatics and Biostatistics, Department 11—Medical Education, Faculty of Medicine, “Iuliu Hatieganu” University of Medicine and Pharmacy, 400349 Cluj-Napoca, Romania; 3 Department of Cardiology, Emergency County Hospital, 430031 Baia Mare, Romania; medicbm@yahoo.com (C.P.); dancorinadelia@yahoo.com (D.M.); 4 Faculty of Medicine Arad, “Vasile Goldis” Western University, 310045 Arad, Romania; 5 Department of Pharmaceutical Biochemistry and Clinical Laboratory, Faculty of Pharmacy, “Iuliu Hațieganu” University of Medicine and Pharmacy, 400012 Cluj-Napoca, Romania; liana.lucaciu@umfcluj.ro; 6 Department of Inorganic Chemistry, Faculty of Pharmacy, “Iuliu Hațieganu” University of Medicine and Pharmacy, 400012 Cluj-Napoca, Romania; adriana.hangan@umfcluj.ro; 7 Department of Immunology, Emergency County Hospital, 400006 Cluj-Napoca, Romania; rodicarahaian@gmail.com; 8 Department of Internal Medicine, “Iuliu Hațieganu” University of Medicine and Pharmacy, 400015 Cluj-Napoca, Romania; angelacozma@umfcluj.com (A.C.); vasile.negrean@umfcluj.ro (V.N.); 9 Department of Medical Biochemistry, “Iuliu Hatieganu” University of Medicine and Pharmacy, 400349 Cluj-Napoca, Romania; lprocopciuc@umfcluj.ro

**Keywords:** heart failure, preserved ejection fraction, FGF21, Galectin3, copeptin, diabetes mellitus

## Abstract

*Background and Objectives*: Heart failure (HF) is one of the most common initial presentations of cardiovascular disease (CVD) in patients with type 2 diabetes mellitus (T2DM). There are different cardiac biomarkers related to the pathophysiological mechanisms of HF in T2DM. The current research aims to identify additional biomarkers that could improve the diagnosis and prognosis of HFpEF, which is currently assessed using NT pro-BNP levels. NT pro-BNP is a valuable tool for diagnosing heart failure but may not always correlate with clinical symptom severity or can present normal levels in certain cases, such as obesity. Biomarkers like FGF-21 and galectin-3 could provide greater insight into heart failure severity, especially in diabetic patients. The main objective of the current study is to assess the performance of NT-proBNP, FGF21, Galectin-3 and Copeptin to discriminate between advanced and mild HF. *Materials and Methods*: A total of 117 patients were enrolled in this study and divided into two groups: 67 patients in NYHA functional class I-II (mild HF) and 50 patients in NYHA III-IV (advanced HF). NT-pro BNP, FGF21, Galectin 3 and Copeptin serum levels were determined with the ELISA method. Receiver operating characteristic (ROC) analysis and binomial logistic regression analysis were used to measure the ability of the studied biomarkers to distinguish between advanced and mild HF patients. *Results*: In patients with T2DM with advanced HF, serum FGF21 level was significantly positively correlated with eGFR (ρ = 0.35, *p* = 0.0125) and triglycerides (ρ = 0.28, *p* = 0.0465) and significantly negatively correlated with serum levels of HDL cholesterol (ρ = −0.29, *p* = 0.0386) and with RV-RA gradient (ρ = −0.30, *p* = 0.0358). In patients with mild HF, serum FGF21 level was significantly negatively correlated with NT-proBNP levels (ρ = −0.37, *p* = 0.0022), E/e’ ratio (ρ = −0.29, *p* = 0.0182), TR velocity (ρ = −0.24, *p* = 0.0470) and RV-RA gradient (ρ = −0.24, *p* = 0.0472). FGF21 (AUC = 0.70, 95% CI: 0.60−0.79) and NT-proBNP (AUC = 0.73, 95% CI: 0.63–0.82) demonstrated significant predictive value to discriminate T2DM patients with advanced HF from those with mild HF. Elevated values for FGF21 (≥377.50 ng/mL) or NTproBNP (≥2379 pg/mL) were significantly associated with increased odds of advanced HF after adjusting for demographic and clinical covariates. *Conclusions*: NTpro-BNP and FGF21 have a similar ability to discriminate T2DM patients with advanced HF from those with mild HF. Univariable and multivariable logistic models showed that, FGF21 and NTproBNP were independent predictors for advanced HF in patients with preserved and mildly reduced ejection fraction and T2DM.

## 1. Introduction

Heart failure (HF) is an increasingly prominent disease in developed countries, placing a significant burden on patients and healthcare systems. It currently affects approximately 64 million people worldwide, with a rising prevalence [1]. In the general population older than 60 years, a percentage of 4.9% were identified to have heart failure with preserved ejection (HFpEF) and this number is expected to increase further as people live longer and obesity and diabetes become more common [2]. Patients with HFpEF constitute nearly 50% of the HF population, which has increased in prevalence in recent years and is now associated with similar mortality rates to heart failure with reduced ejection fraction (HFrEF) [2,3,4]. Associated comorbidities such as hypertension, diabetes mellitus, atrial fibrillation, obesity, chronic kidney disease and non-cardiovascular comorbidities are more frequent in HFpEF than in patients with HFrEF. However, they do not fully explain the impaired prognosis, as higher mortality rates have been observed in patients with HFpEF compared to those with similar comorbidities but without HF [5].

The major etiological factors causing HF in T2DM include hypertension, ischemic heart disease, the direct and indirect impact of hyperglycemia, and obesity [6]. Coronary artery disease in T2DM patients is frequently characterized by severe, diffuse lesions and can often be asymptomatic [6].

Diabetes mellitus (DM) has an estimated global prevalence of 537 million cases, representing a growing burden on healthcare systems [1]. In patients with T2DM, HF is one of the most common initial presentations of cardiovascular disease (CVD) [6]. HF and T2DM are two interconnected pathologies; patients with T2DM have an approximately two-to-four-fold increased risk of developing HF compared to those without this metabolic disease [1,6,7]. Moreover, patients who suffer from HF have T2DM as an associated comorbidity, with rates ranging from 30% to 40%, or increasing to high as 50% among patients hospitalized for HF [7].

A recent updated meta-analysis (Hoek et al., 2024) reported prevalences of 43% for left ventricle dysfunction and 17% for HFpEF in patients with T2DM [7,8], and lower prevalences of 6% and 7% for left ventricle systolic dysfunction (LVSD) and HFrEF [9]. The incidence of HFpEF in diabetic patients ranged from 2.5% to 20.8% in the hospitalized population and from 4.2% to 8.9% in the general population [7].

A significantly elevated risk of all-cause mortality, cardiovascular disease-related mortality and initial hospitalization for decompensated HF are observed in patients with both T2DM and HF [1]. Patients included in the RELAX trial with HFpEF and concomitant T2DM exhibited an increased risk of hospitalization for HF (47%) compared to those without diabetes (28%). Additionally, they showed a higher likelihood of hospitalization for cardiac (23.7%) or renal (4.9%) complications at 6 months [10].

Long-term outcomes are also poor, as T2DM emerged as a predictor of both all-cause mortality and hospitalization risk for HFpEF with a hazard ratio of 1.72 (95% confidence interval 1.1, 2.6) over a duration of 25 ± 11 months. This association remained significant regardless of age, body mass index (BMI), renal function, and functional classification [11].

According to the European Society of Cardiology (ESC) Heart Failure Guidelines, HF is classified into three categories according to left ventricle ejection fraction (LVEF): HF HFrEF defined as LVEF ≤ 40%; HF with mildly reduced ejection fraction (HFmrEF), where LVEF ranges from 41% to 49%; and HFpEF characterized by LVEF ≥ 50% [3].

However, the optimal identification of HFpEF remains challenging, and studies relate that many patients reporting dyspnea are underdiagnosed [4,8].

Multiple non-invasive diagnostic algorithms have been proposed to facilitate the correct diagnosis of HFpEF, including the H2FPEF [12] and HFA-PEFF [4] scores, but they have the limitation of patients being classified discordantly [13,14,15]. However, in instances where HF is suspected and the above-mentioned scores place the patient in an intermediate category, a correct diagnosis requires additional diastolic stress testing [16], including exercise echocardiography and/or (exercise) right heart catheterization [17] to assess pulmonary capillary wedge pressure, considered to be ‘the gold standard’ [16,17,18].

Natriuretic peptides (NPs), such as brain (B-type) natriuretic peptide (BNP) and N-terminal prohormone of brain natriuretic peptide (NT-proBNP), are useful for establishing the diagnosis of HFpEF, but due to the high specificity and very high negative predictive value (94–97%) of NT-proBNP, it is likely that NT-proBNP is more suitable for ruling out HFpEF [19,20]. Moreover, elevated levels of BNP and NT-proBNP are markers of HF severity and prognosis, being associated with an augmented risk for adverse short- and long-term outcomes in HF, including all-cause and cardiovascular mortality [19]. A high left ventricular end-diastolic wall stress is considered the main trigger for the release of NPs, which is inversely proportional to wall thickness. Therefore, the excellent negative predictive value of natriuretic peptides is applicable, particularly in HFrEF, characterized by a dilated left ventricle, but not necessarily in HFpEF, where left ventricular hypertrophy tends to normalize wall stress [4,21]. In consequence, there are studies that proved the existence of HFpEF by invasive hemodynamic measurement of LV filling pressures in up to 20–30% of patients that had NPs below the diagnostic cut-offs [4,18,22]. Moreover, NP levels are often normal in obese patients with HFpEF and in patients with normal resting left ventricle filling pressures that become elevated with exercise [7,19], and tend to have higher values in elderly populations, with various cardiac (acute coronary syndrome, valvular heart disease, arrhythmias, pulmonary hypertension) or non-cardiac causes (altered kidney function, anemia, sepsis, pulmonary disease, liver disease, ischemic or hemorrhagic stroke) [3,19].

Given that HFpEF in T2DM patients is often underdiagnosed and that there is also a paucity of treatment options with convincing evidence for minimizing cardiovascular outcomes [6], the emergence of novel cardiac biomarkers to enhance the diagnosis, prognosis and therapeutic assessment of HF has gathered interest. There are various cardiac biomarkers related to the pathophysiological mechanisms of HF in T2DM: biomarkers of myocardial stretch (natriuretic peptides, inflammation and fibrosis (galectin-3, fibroblast growth factors, interleukin-6, tumor necrosis factor α, suppression of tumorigenicity 2, transforming growth factor β, cardiotrophin 1), the neuroendocrine process (copeptin), extracellular matrix remodeling and microRNAs [23,24].

NT pro BNP is a biologically inactive molecule resulting from pro-BNP cleavage, and it is expressed by ventricular cardiac myocytes in response to increased ventricular wall stretch [19].

Gal-3 is a soluble β-galactoside-binding lectin, ubiquitously distributed in various tissues, located in the cytoplasm and the extracellular space, its expression being upregulated in injured tissues that are undergoing remodeling processes. It is primarily produced by infiltrating macrophages as well as a subset of activated fibroblasts and vascular cells, functioning as a biomarker for inflammation and fibrosis [25]. In healthy cardiac tissue, Gal-3 baseline expression is very low, with a rapid increase in cases of myocardium injury for tissue repair, but its sustained overexpression has a role in the pathogenesis of cardiac fibrosis hypertrophy and dysfunction [26].

Fibroblast growth factor 21 is a polypeptide that plays an important role in the modulation of glucose homeostasis and lipid metabolism, interacting with FGF and Beta Klotho receptors, which results in elevated blood glucose concentrations and the induction of ketogenesis. There is evidence indicating that cardiac myocytes secrete FGF21 as an autocrine signaling molecule to mitigate adverse cardiac remodeling processes [27,28]. The cardiac expression of FGF21 is modulated by the protein deacetylase Sirt1 (sirtuin1), especially in the presence of high glucose and lipidic levels, in order to shield cardiomyocytes from oxidative stress by enhancing the expression of specific antioxidant genes [29].

Copeptin represents a fragment of pre-provasopressin. Arginine vasopressin (AVP) is produced in the hypothalamus as a response to conditions of hypovolemia. Elevated levels of AVP in heart failure are considered to be a factor in the advancement of left ventricular remodeling by promoting the hypertrophy of cardiomyocytes. However, because AVP is unstable and has a short half life, copeptin is secreted in equimolecular quantities corresponding to AVP and is stable, which makes it a good surrogate biomarker for diagnosis and prognosis in heart failure [30,31].

The objectives of the current study were (i) to compare the clinical and paraclinical features between patients with advanced HF and those with mild HF (by NYHA classification); (ii) to compare the distributions of NT-proBNP, FGF21, Galectin-3 and Copeptin between the studied groups; (iii) to assess the performance of NT-proBNP, FGF21, Galectin-3 and Copeptin to discriminate between advanced and mild HF; and (iv) to evaluate the potential correlations between the studied biomarkers, echocardiographic and clinical parameters in T2DM patients stratified by the severity of HF.

## 2. Materials and Methods

### 2.1. Study Design and Characteristics of the Groups

Between February 2019 and February 2024, a total of 117 patients with T2DM and HF that fulfilled the inclusion criteria were enrolled in this study at the ‘’Niculae Stăncioiu’’ Heart Institute Cluj-Napoca, Romania, and at the ’’Dr Constantin Opriș’’ Emergency County Hospital, Baia Mare, Romania. Of these, 67 patients had mild HF, with established chronic HF without changes in symptoms and signs in the last month, or de novo HF (NYHA classes I or II), and 50 patients had advanced HF, with chronic worsening HF or with de novo acute HF (NYHA classes III or IV). Patients were divided into these two groups according to the severity of symptoms using the New York Heart Association (NYHA) functional Classification [3].

This study was conducted according to the guidelines of the Declaration of Helsinki, and approved by the Ethics Committee of Iuliu Hatieganu University of Medicine and Pharmacy, Cluj-Napoca, Romania (protocol code 48/11 March 2019) and Emergency County Hospital Baia Mare, Romania (no. 6361/28 February 2020). All subjects included in this study provided informed consent.

The inclusion criteria consisted of the presence of T2DM (under treatment or newly diagnosed), heart failure with preserved ejection fraction (HFpEF) and mildly reduced ejection fraction (HFmrEF) with a left ventricular ejection fraction (LVEF) ≥ 40% defined according to the European Society of Cardiology (ESC) Heart Failure Guidelines 2021 [3], and age over 18 years old. The exclusion criteria were severe valvular disease, infiltrative or hypertrophic cardiomyopathy, congenital heart disease, pericardial disease, acute coronary syndrome, acute pulmonary embolism, atrial fibrillation or flutter with high ventricular response > 100 bpm, supraventricular or ventricular tachycardia, cardiac surgery, chronic obstructive pulmonary disease GOLD stages 3 or 4, pneumonia, COVID-19 infection, ischemic or hemorrhagic stroke, severe renal failure (eGFR by CKD-EPI < 30 mL/min/1.73 m^2^), severe anemia (hemoglobin < 7 g/dL), liver cirrhosis, neoplasia, sepsis or thyrotoxicosis.

We defined HFpEF and HFmrEF according to the recommendations of the ESC Guidelines 2021 [3]. For patients with chronic HFpEF, the diagnosis algorithm HFA-PEFF was used, recommended by the Heart Failure Association (HFA) [4] based on clinical examination revealing signs and symptoms of HF, echocardiographic parameters of function and morphology and the level of serum natriuretic peptides. A HFA-PEFF score ≥ 5 points confirmed the diagnosis of HFpEF, and for patients with a score between 2 and 4, exercise echocardiography was performed.

The diagnosis of HFmrEF required the presence of symptoms and/or signs of HF, and an echocardiographic measurement of LVEF between 40 and 49%. For patients presenting with acute decompensated HF (chronic worsening HF or ‘new onset’ acute HF), we used higher NT pro BNP cut-off values related to age: <55 years: NTproBNP > 450 pg/mL, 55–75 years: >900 pg/mL, and >75 years: >1800 pg/mL.

### 2.2. Biomarker Analysis

Blood samples were collected at the time of admission in serum separator tubes with a clot activator (BD Vacutainer CAT) and permitted to clot at room temperature for no more than 2 h. Afterwards, they were centrifugated for 15 min at 1000× *g* to obtain serum. The samples were isolated into Eppendorf tubes and stored at −80 °C until they were analyzed. The NT-proBNP analysis was carried out by an electrochemiluminescent immunoassay (Roche Diagnostics, Mannheim, Germany). The serum concentration of the biomarkers were assessed through a quantitative enzyme-linked immunosorbent assay (ELISA): FGF21(Catalog No E-EL-H0074, sensitivity 18.75 pg/mL, detection range: 31.25–2000 pg/mL, Elabscience Biotechnology Inc., Houston, TX, USA), Galectin-3 (Catalog No E-El-H1470, sensitivity 0.1 ng/mL, detection range 0.16–10 ng/mL, Elascience Biotechnology Inc., Houston, TX, USA), Copeptin (Catalog No E-EL-H0851, sensitivity 18.75 pg/mL, detection range 31.25–2000 pg/mL, Elabscience Biotechnology Inc., Houston, TX, USA).

### 2.3. Echocardiographic Assessment

A complete two-dimensional, M-mode and Doppler transthoracic echocardiography evaluation was performed using a cardiology software application (Philips CX 50, xMATRIX, version 5.0.2, Philips Ultrasound Inc., Bothell, WA, USA). LVEF was evaluated by using Simpson’s method. Left ventricle end-diastolic volume (LVEDV) and left ventricle end-systolic volumes (LVESV) were identified using the apical 4-chamber view. Transmitral inflow, the peak velocities of early (E) and late (A) diastolic filling waves and E deceleration time (EDT) were measured from the apical 4-chamber view using pulsed wave blood flow Doppler between the mitral valve leaflets in diastole. In order to calculate the E/e’ ratio, we assessed early diastolic mitral annulus velocity (e’) with a pulsed wave tissue Doppler placed on the LV septum and lateral wall. Pulmonary arterial systolic pressure was obtained using the modified Bernoulli equation as 4× tricuspid regurgitation velocity plus the addition of the estimated right atrial pressure. The global longitudinal strain of the left ventricle was analyzed using 2D speckle tracking echocardiography and automated functional imaging software (AFI) version 5.0.2

### 2.4. Statistical Analysis

Demographic and clinical features which followed a Gaussian distribution were described using mean and standard deviation. Non-parametric distributions of clinical and echocardiographic features were summarized by median and interquartile range (IQR = [25th percentile; 75th percentile]). The Gaussian distribution in each studied group was assessed by several methods: quantile–quantile plots and Shapiro–Wilk tests.

Comparisons of the clinical and paraclinical features, echocardiographic parameters and studied biomarkers in T2DM patients with advanced HF and those with mild HF were performed using Student’s *t*-test with equal variances, Welch’s *t*-test, Chi-square test or Fisher’s Exact test.

Tests for the correlations between biomarkers (FGF21, NTproBNP, Galectin-3, Copeptin) and echocardiographic features were performed using non-parametric Spearman’s correlation coefficient and significance tests.

To evaluate the ability of each biomarker to discriminate T2DM patients with advanced HF from those with mild HF, ROC analysis was used to assess the sensitivity and specificity of these biomarkers. The ROC analysis provided an AUC (area under the curve) metric for each biomarker and 95% confidence interval for AUC as well as an optimal cut-off value estimated based on the Youden index. To determine if the AUCs of significant biomarkers were significantly different from each other, we used the DeLong test to compare the multiple ROC curves.

Based on the optimal cut-off valuers estimated by ROC analysis, we tested two multivariable logistic models to test if the biomarkers remained significant predictors for advanced HF in patients with preserved and mildly reduced ejection fraction and type 2 diabetes mellitus.

All statistical tests used in the current study were two-sided statistical tests with a significance level α = 0.05. All statistical analyses were conducted using R software, version 4.4.0 (R Foundation for Statistical Computing, Vienna, Austria).

## 3. Results

### 3.1. Characteristics of the Studied Sample of T2DM Patients

A sample of 117 patients meeting the inclusion criteria were divided into two groups based on symptomatic and physical activity severity according to NYHA functional class. Patients with mild HF (NYHA classes I or II, n = 67) and patients with advanced HF (NYHA classes III or IV, n = 50). The distributions of baseline demographic, anthropometrics and clinical features by group type are described in Table 1. Regarding baseline characteristics, in our study that included T2DM patients with HFpEF and HFmrEF, we identified a median age of 67 years, of whom 52% were female, 61.53% were obese, 84% were hypertensive, 52% had myocardial infarction, 32.4% had three-vessel coronary lesions and 26% had atrial fibrillation. The term atrial fibrillation included persistent atrial fibrillation (that lasted more than 7 days), long-standing persistent atrial fibrillation with a duration of at least 12 months but where rhythm control was still a treatment option (but the patient temporized the procedure), or permanent atrial fibrillation where there were no further attempts to restore the sinus rhythm. The term paroxysmal atrial fibrillation referred to episodes that lasted <7 days, that terminated spontaneously or that needed medication.

Patients with advanced HF had significantly different body mass index (BMI) values than patients with mild HF (*p* = 0.00095) and body surface area (BSA) (*p* = 0.01251).

We noticed that patients with advanced HF had a significant proportion of atrial fibrillation (*p* = 0.00021) and significant differences in the proportions of obesity (*p* = 0.0167) and chronic obstructive pulmonary disease (COPD) (*p* = 0.0001) than those with mild HF.

The frequency of metformin treatment was higher in patients with mild HF than in those with advanced HF (83.6% vs. 68.0%) with marginal statistical significance. Regarding insulin treatment, it was more frequent in patients with advanced HF than in those with mild HF (66.0% vs. 47.8%) (Table 2).

Patients with advanced HF had lower glomerular filtration rates (eGFR, mL/min/1.73 m^2^) than patients with mild HF (median [IQR]: 69.5 [46.25, 86.75] vs. 83.0 [59.0, 101.0], *p* = 0.01226) and higher urea levels (median [IQR]: 53.59 [38.49, 73.60] vs. 38.95 [30.32, 54.50]). Also, uric acid levels were higher in those with advanced HF (mean (SD): 7.25 (2.12) vs. 6.29 (1.58)) (Table 2).

Serum FGF21 levels and NT-proBNP levels were significantly different in patients with advanced HF compared to patients with mild HF (Table 3). Regarding Copeptin (*p* = 0.7745) and Galectin-3 (*p* = 0.3327), we found no significant difference between the studied groups.

It was noteworthy that the echocardiographic parameters such as LAVi, LA dimension, LA surface, TR velocity, RV-RA gradient and PASP were significantly different between HF groups (Table 3). T2DM patients with advanced HF tended to have higher values than those with mild HF.

### 3.2. Correlations Between FGF21, NT-proBNP, Galectin-3 and Copeptin with Clinical and Echocardiographic Features

Elevated serum FGF21 levels were significantly correlated with elevated eGFR (ρ = 0.35, *p* = 0.0125) and triglycerides (ρ = 0.28, *p* = 0.0465) and decreased serum levels of HDL cholesterol (ρ = −0.29, *p* = 0.0386) and values of RV-RA gradient (ρ = −0.30, *p* = 0.0358) in patients with advanced HF.

In patients with mild HF, elevated serum FGF21 levels were significantly correlated with decreased NT-proBNP levels (ρ = −0.37, *p* = 0.0022), E/e’ ratio (ρ = −0.29, *p* = 0.0182), TR velocity (ρ = −0.24, *p* = 0.0470) and RV-RA gradient (ρ = −0.24, *p* = 0.0472). In this group, Galectin-3 level was significantly negatively corelated with Copeptin level (ρ = −0.32, *p* = 0.0078) (Table 4).

Elevated serum NT-proBNP levels were significantly correlated with advanced age (ρ = 0.31, *p* = 0.0273), elevated levels of urea (ρ = 0.29, *p* = 0.0401), uric acid (ρ = 0.30, *p* = 0.0338), TR velocity (ρ = 0.38, *p* = 0.0068), RV-RA gradient (ρ = 0.31, *p* = 0.0301) and PASP (ρ = 0.39, *p* = 0.0053) only in T2DM patients with advanced HF. In the same group, NT-proBNP was significantly negatively correlated with BMI (ρ = −0.32, *p* = 0.0232), total cholesterol (ρ = −0.32, *p* = 0.0247) and LDL cholesterol (ρ = −0.29, *p* = 0.0379) (Table 4).

In patients with mild HF, elevated serum levels of galectin 3 were negatively correlated with serum copeptin levels (ρ = −0.32, *p* = 0.0078) and also hemoglobin (ρ = −0.29, *p* = 0.0154) and positively correlated with BMI (ρ = 0.46, *p* = 0.0001). In the same group, copeptin levels were negatively correlated with BMI (ρ = −0.25, *p* = 0.0417).

### 3.3. Distinguishing Between Patients with Moderate-to-Severe HF and Those with Mild HF

**Figure 1:**
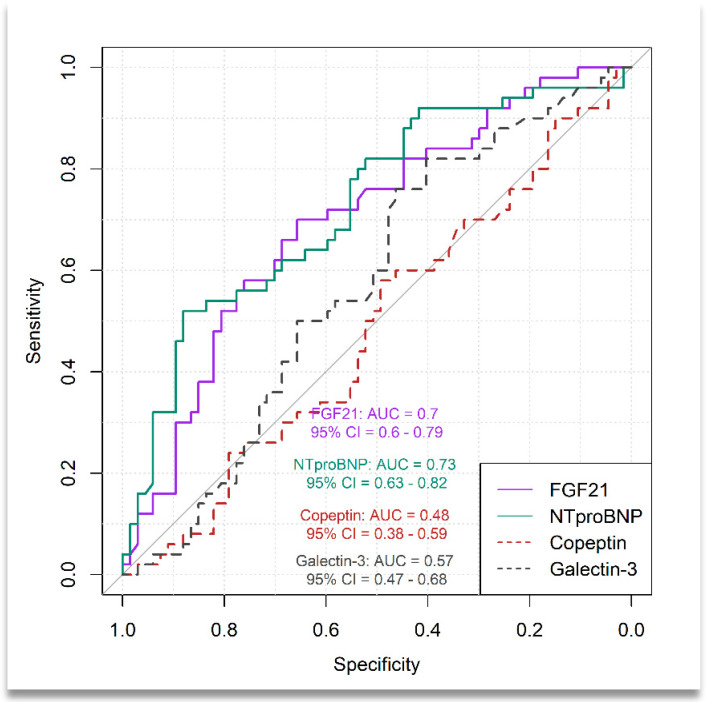
ROC plot of serum levels of FGF21 and NT-proBNP for predicting the presence of advanced HF in patients with T2DM.

As shown in Figure 1, the receiver operating characteristic (ROC) analysis indicated an estimated value of area under the curve for serum FGF21 level predicting advanced HF equal to 0.70 (95% CI: 0.60–0.79) and an optimal cut-off value based on the Youden index of 377.50 ng/mL, with a sensitivity of 70% (95% CI: 55.39–82.14%), specificity of 65.67% (95% CI: 53.06–76.85%), PPV of 60.34% (95% PPV: 47.35–74.99%) and NPV of 74.58% (95% CI: 60.95–83.58%).

The performance of serum NT-proBNP level in discriminating between patients with advanced HF and those with mild HF was as follows: AUC = 0.73 (95% CI: 0.63–0.82), optimal cut-off value = 2379 pg/mL, Se = 52.0% (95%CI: 37.42–66.34%), Sp = 88.06% (95%CI: 77.82–94.70%), PPV = 76.47% (95% CI: 60.73–85.53%) and NPV = 71.08% (95% CI: 57.57–85.63%).

The results of DeLong’s test suggested that there was no significant difference between FGF21 and NT-proBNP in distinguishing between patients with advanced HF and those with mild HF (*p* = 0.7052, 95% CI for difference in AUC: −0.18 to 0.12), but FGF21 had moderate sensitivity and specificity while NT-proBNP had high specificity but low sensitivity.

The results also showed that Galectin-3 and Copeptin were not able to distinguish advanced HF from mild HF (Galectin-3: AUC = 0.57, 95%CI: 0.47–0.68, Copeptin: AUC = 0.48, 95% CI: 0.38–0.59).

### 3.4. FGF21 and NT-proBNP as Independent Predictors for Advanced HF in T2DM Patients

Univariable and multivariable logistic models showed that after adjusting for other known risk factors, FGF21 (≥377.50 ng/mL vs. <377.50 ng/mL) and NT-proBNP (≥2379 pg/mL vs. <2379 pg/mL) were independent predictors for advanced HF, with elevated values of FGF21 or NTproBNP being significantly associated with increased odds of advanced HF in all tested models (Table 5).

## 4. Discussion

Regarding baseline characteristics, in our study that included T2DM patients with HFpEF and HFmrEF, the findings are in agreement with the results from some clinical trials, such as PARAGON-HF, PARAMOUNT and PARAGLIDE-HF, which included patients with T2DM as almost half of the samples [32,33,34].

We also observed that patients with advanced HFpEF have a significant burden of comorbidities compared to those with mild HF. In this group, there is a higher prevalence of obesity, atrial fibrillation, chronic obstructive pulmonary disease (COPD), chronic kidney disease and an increased use of insulin treatment. All of these comorbidities can worsen HF. AF and HF often coexist and exacerbate each other, with their co-occurrence increasing with age and HF severity, leading to poorer outcomes like higher stroke risk and mortality. Obesity contributes significantly to HFpEF and presents different pathophysiologic mechanisms than in non-obese HFpEF patients. CKD often coexists with HF, sharing risk factors such as diabetes and hypertension, and can worsen cardiovascular function through mechanisms like oxidative stress and fibrosis. CKD is more common in HFpEF but impacts prognosis less severely than in HFmrEF and HFrEF. COPD, present in about 20% of HF patients, significantly affects symptoms and outcomes, and differentiation between HF and COPD can be challenging due to overlapping symptoms; proper COPD management can improve cardiac function. There is a stronger association between obesity and HFpEF. In obese patients diagnosed with HF, an obesity paradox has been documented, indicating that patients with overweight or obesity grade I or II exhibit a more favorable prognosis in comparison to underweight individuals. Nonetheless, the obesity paradox is not evident among patients suffering from T2DM, due to other factors that may modulate this association [35]. The findings in our study are in concordance with this statement, given the fact that the group with advanced HF demonstrated a higher prevalence of obesity. Adipose tissue significantly influences the diagnostic and prognostic value of various parameters. Obese patients who are suffering from HF exhibit diminished concentrations of NPs due to the heightened expression of clearance receptors and the enhanced degradation of peptides by adipose tissue [19]. In our study, NT-proBNP values were inversely correlated with BMI, as stated in the literature [36,37].

Regarding the paraclinical parameters, patients with T2DM and advanced HF tended to have lower glomerular filtration rates (eGFR, mL/min/1.73 m^2^) and higher urea and uric acid levels than patients with mild HF, consistent with previous studies. In a cohort of 8875 patients with HFpEF and 8374 with HFmrEF (half of whom had T2DM), the prevalence of chronic kidney disease (CKD) was 56% in HFpEF and 48% in HFmrEF, particularly among those in NYHA classes III and IV. The one-year mortality rates were significantly higher for patients with CKD: 23% in HFpEF, 22% in HFmrEF and 23% in HFrEF, compared to 13%, 8% and 8% in those without CKD (*p* < 0.001 for all).

After adjustments, CKD showed a stronger association with mortality in HFrEF and HFmrEF compared to HFpEF, with hazard ratios of 1.49 and 1.51, respectively, versus 1.32 for HFpEF (P for interaction < 0.001). Receiver operating characteristic (ROC) analyses also indicated that CKD was a better predictor of death in HFrEF and HFmrEF (AUC of 0.699 and 0.700) than in HFpEF (AUC of 0.629). Although CKD is more prevalent in HFpEF, it is associated with a lower mortality risk and prognostic discrimination in this group [38]. Regarding hyperuricemia, it may be caused or aggravated by diuretic treatment and it is related to symptoms, exercise capacity, the severity of diastolic dysfunction and long-term prognosis. For every 1 mg/dL increase in serum uric acid levels, the risks of all-cause mortality and of HF hospitalization increase by 4% and 28%, respectively [39].

There was a large percentage of patients with high levels of HbA1c that did not meet the glycemic target in order to reduce T2DM complications (76% of patients with HbA1c > 7%), but there were no differences in HbA1c mean between the two groups. This result can be explained by the fact that we enrolled both patients with a long history of T2DM and also newly diagnosed patients with high values of HbA1c in this study.

The guidelines recommend tight glycemic control (HbA1c < 7%) to reduce microvascular complications, but studies failed to demonstrate an impact of intensive glycemic control on short- or medium-term macrovascular complications (over 3.5–10 years) [1]. A meta-analysis revealed that reducing HbA1c lowers major adverse cardiovascular events (MACEs) mainly by reducing myocardial infarction, but without a significant effect concerning HF (Zoungas 2017) [40], and reduces microvascular complications such as nephropathy and retinopathy (Turnbull 2009) [41].

The guidelines for the management of CV disease in T2DM patients maintain the same recommendation as in the general population for measuring BNP or NT-proBNP for diagnosis or prognosis assessments in the management of both HFrEF and HFpEF. Natriuretic peptides such as brain (B-type) natriuretic peptide (BNP) and N-terminal prohormone of brain natriuretic peptide (NT-proBNP) are considered useful biomarkers to reveal the presence and severity of HF. Although they proved to have a correlation with NYHA class, an important overlap in NP concentrations was observed among the NYHA classes [19].

A frequent misconception is that levels of BNP or NT-proBNP predominantly reflect congestion across all physiological domains. While this conception is validated in cases of severe, advanced HF or in decompensated HF, where congestion serves as the principal factor influencing the elevation of natriuretic peptides, in chronic stable HF, the primary determinant of BNP or NT-proBNP levels is transmural wall stress. This stress is determined more significantly by cardiac structural and functional parameters, including the dimensions of the left atrium or left ventricle, valvular abnormalities, or cardiac rhythm. This illustrates the robust correlation between BNP and NT-proBNP and cardiac remodeling, which is strongly connected to clinical outcomes [19].

In our study, NT-pro BNP levels correlated with advanced age and the severity of heart failure, being significantly elevated in the group of patients with advanced HF.

A study conducted by Lebedev et al. in 2020 that investigated the profile of variate biomarkers (NT-pro BNP, galectin-3, ST2, MMP-9, hs-CRP, PIIINP, TIMP-1) in 62 patients with diabetes and obesity and chronic stable HF with preserved and midrange EF (in the NYHA II functional class and optimal HF drug therapy for a minimum of 3 months) and diabetic patients without HF. They obtained lower levels of NT pro BNP in patients with HFpEF and HFmEF (133.6 pg/mL and 162.5 pg/mL, respectively) compared to our study [42].

In our study, the levels of NT pro BNP were higher even if patients had mild symptoms of HF. This highlights the importance of evaluating NP levels periodically in all patients with HFpEF/HFmrEF irrespective of the severity of clinical presentation, because they are valuable in establishing a prognosis.

In our study, serum NT-proBNP levels had high specificity (88.06%) but low sensitivity (52%) when discriminating between T2DM patients with advanced HF from those with mild HF at an optimal cut-off value of 2379 pg/mL. This observation confirmed that NTproBNP could be used for diagnosing advanced HF.

A biomarker that raises interest is FGF21. FGF21 is a peptide hormone that plays a role in lipid and glucose metabolism and preservation of energy balance. Its concentration is elevated in the early stages of multiple metabolic disorders and is regarded as a hormone that is activated as a compensatory response in reaction to stress. Results from prior investigations have demonstrated that FGF21 is significantly elevated in individuals diagnosed with obesity, insulin resistance, T2DM, dyslipidemia and non-alcoholic fatty liver disease [43]. Higher FGF21 levels might represent a compensatory response to intrinsic metabolic stress or may be due to dysfunctional FGF21 signaling, resulting in FGF21 resistance [44]. Studies relate FGF21 resistance in patients with obesity to the release of pro-inflammatory molecules and microRNAs, leading to an increase in FGF21 plasma levels and decreased expression of the FGF21 receptor complex [45]. FGF21 resistance causes various metabolic disturbances like increases in blood glucose, circulating fatty acids, oxidative stress and inflammation, decreases in insulin sensitivity and lipolysis. Preclinical and clinical studies with FGF21 analogs and FGF 21 receptor agonists show improvements in glycemic and lipidic profiles, insulin sensitivity and reduced body weight [45].

High serum levels of FGF21 are correlated with diabetic cardiomyopathy (DCM); nonetheless, it is not clear whether increased levels contribute to DCM pathogenesis or are involved in repairing injury caused by DCM. FGF21 protects cardiomyocytes against oxidative stress, inflammation and hypertrophy [44]. Additionally, Lenart-Lipińska et al. provided evidence of increased FGF21 levels in patients suffering from ischemic coronary heart disease, chronic kidney disease and in those with a detrimental lipid profile [46]. In our study, we found that serum FGF-21 correlated with eGFR, elevated levels of triglycerides and low levels of HDL cholesterol in patients presenting with advanced HF. We confirmed the fact that FGF21 has a possible implication in lipidic metabolism. Also, FGF21 was correlated with RV-RA gradient. Moreover, in patients with mild HF, serum FGF21 level was significantly negatively correlated with NTproBNP levels, E/e’ ratio, TR velocity and RV-RA gradient.

In a study conducted by Chou et al. in 2016, with a percentage of 34% T2DM patients, FGF21 was non-inferior to NT pro BNP in diagnosing diastolic dysfunction and predicting cardiovascular events over a 1-year follow up [27]. In a previous study on a sample of patients with T2DM, we demonstrated that FGF21 has a moderate performance in diagnosing HFpEF and is non-inferior to NT proBNP [47]. Another study performed by Ong et al. in 2015 [44] showed that patients with T2DM with established microvascular complications have higher baseline concentrations of FGF21, which served as a prognostic indicator for the future emergence of novel microvascular complications during a 5-year follow up [48]. Lenart-Lipinska et al., 2013 [43] obtained a median value of FGF21 = 240.7 pg/mL in patients with T2DM and demonstrated that FGF21 levels above the median value correlated with an increased incidence of CV events such as heart failure, myocardial infarction, stroke and coronary revascularization during a 2-year period of follow up, with an HR of 4.7 [43].

As far as we know, there are no studies that have compared FGF21 levels according to the severity of HF symptoms. Our findings indicate a median value of FGF21 = 322 pg/mL in T2DM patients with mild symptomatic HF, with a significantly higher value of 580 pg/mL in T2DM patients with advanced HF. FGF21 has a high sensitivity (70%) and specificity (65.67%) in identifying advanced HF in T2DM at an optimal cut-off value of 377.50 ng/mL. This observation suggests that FGF21 could be used to discriminate T2DM patients with advanced HF from those with mild HF. Using multivariate analysis, we confirmed in the present study that FGF21 and NTproBNP were independent predictors for advanced HF.

Galectin-3 (Gal-3) is a beta-galactoside-binding lectin with roles in inflammation, fibrosis processes, causing cardiac remodeling by increasing myofibroblast proliferation, the extracellular matrix and macrophage infiltration [48]. In the general population, elevated Gal-3 serum levels were reported to be positively associated with age, T2DM, obesity, hypercholesterolemia, hypertension and target organ injury [49,50]. Previous studies showed that Gal-3 is correlated with T2DM prevalence and incidence, potentially via the inflammatory signaling cascade that causes β-cell fibrosis and hinders insulin secretion [51], and was also shown to be higher in patients with obesity in a study by Weighert et al. [49,52]. Gal-3 exhibits both pro-inflammatory and anti-inflammatory properties, depending on the setting and specific target cell or tissue. Obesity and T2DM are conditions characterized by chronic inflammation in which Gal-3 acts for promoting repair and to limit tissue injury [49]. Gal-3 is also responsible for the initiation and advancement of T2DM complications because of its capacity to bind the advanced lipoxidation and glycation products that accumulate in target organs [51]. Gal-3 was also shown to be elevated in left ventricular diastolic dysfunction and in heart failure and was correlated with adverse cardiovascular events and mortality [53,54,55]. In our study, we reported a medium value of Gal-3 in patients with T2DM and HF of 12.46 ng/mL. This result is in concordance with a study conducted by Lebedev et al. in 2020 [55] that stated a medium Gal-3 value of 11.7ng/mL and 10.4ng/mL in patients with HFPEF/HFmEF and T2DM and were significantly higher comparative to T2DM patients without HF in whom the value of Gal-3 was 8.6 ng/mL (*p* < 0.05) [55]. The TOPCAT trial also reported higher values of Gal-3 in patients with T2DM and HFpEF (22 ng/mL) in comparison with T2DM patients without HF (20 ng/L, *p* < 0.001) [56]. Even though in our study Gal-3 was not able to distinguish advanced HF from mild HF, the obtained values are useful in stratifying the risk. The AHA/ACC Guidelines recommend Gal-3 as a biomarker of prognosis in HF (level of evidence IIB), being associated with major acute cardiovascular events and mortality. Also, in patients with HFpEF in whom fibrosis is predominant, objectivated by increased levels of Gal-3, therapeutic targeting holds future interest. An evidence-based algorithm including Gal-3 ± NT pro BNP levels has been proposed for risk stratification. Patients with acute or worsening HF with a Gal-3 level > 17.8 ng/mL, or a Gal-3 level between 9.5 and 17.8 ng/mL and NT pro BNP ≥ 2000 are at high risk of rehospitalization and death. Patients with Gal-3 levels between 9.5 and 17.8 ng/mL and NT pro BNP levels 500–2000 pg/mL are at intermediate risk. Those with a Gal-3 level <9.5 ng/mL and NT pro BNP between 500 and 2000 pg/mL in the acute setting are considered to be at low risk. For patients with chronic stable HF, a Gal-3 level > 25.9 ng/mL places them at high risk.

In HF, a low cardiac output that mimics a hypovolemic condition stimulates the baroreceptors, which increases the levels of a hormone with antidiuretic and vasoconstrictive effects named vasopressin. Copeptin derives from its precursor, pro-vasopressin, secreted in the hypothalamus, which is unstable and has a low half life [57]. A recent meta-analysis by Zimodro et al., 2022 reports higher copeptin levels in patients with HF in comparison with those without [31]. Another meta-analysis that enrolled 4473 patients with chronic and acute HF showed that copeptin predicted mortality from all causes (RR = 2.64) with a comparable performance to NT-proBNP [30]. A study that included 268 patients with severe HF in NYHA functional classes III-IV, 42% with T2DM, demonstrated that copeptin is a good predictor of rehospitalizations and mortality, with a superior value in comparison to BNP [57]. In our study, Copeptin did not show success in distinguishing patients with advanced HF from those with mild HF. A future study with a larger population is needed for a better clarification.

To the best of our knowledge, this is the first study to evaluate the levels of multiple biomarkers according to HF functional severity assessed by NYHA class in T2DM patients with HFpEF and HFmrEF. The present study has several limitations. First, the relatively small sample size. Second, each group included both patients with chronic established HF and ‘de novo’ HF. We obtained data about the levels of serum biomarkers only at the time of admission, and no information was obtained about the changes in their concentrations at the time of discharge or readmission for worsening heart failure.

## 5. Conclusions

The biomarkers NT-proBNP and FGF-21 can be used to discriminate T2DM patients with advanced HF (NYHA class III-IV) from patients with mild HF (NYHA class I-II). The results of our study suggest that using a combination of two biomarkers (NT-proBNP and FGF21) could enhance the diagnostic accuracy for advanced HF. In diabetic patients, we may consider FGF21 as a primary biomarker due to its relevance in metabolic dysregulation, especially in patients with NT pro BNP within the normal range. FGF21 levels higher than 377.50 ng/mL and NT-proBNP levels over 2379 pg/mL can serve as independent predictors for advanced HF.

This study provides ideas for further studies to detect biomarkers of severity in diabetic patients with HFpEF/HFmrEF and discover novel therapeutic agents that can mitigate the risk of worsening heart failure and improve prognosis.

## Figures and Tables

**Table 1:** Demographic, anthropometric and clinical features stratified by HF severity.

	Mild HF (n = 67)	Advanced HF (n = 50)	*p*-Value
Demographic characteristics			
Age (years) ^a^	66.61 (9.89)	67.56 (11.02)	0.6261
Male ^b^	34 (50.7)	23 (46.0)	0.6114
Antropometrics			
Body mass index (kg/m^2^) ^a^	30.90 (5.60)	34.73 (6.62)	0.00095 *
Body surface area (m^2^) ^a^	1.96 (0.21)	2.06 (0.20)	0.01251 *
Clinical characteristics			
SBP (mmHg) ^c^	135 (125 to 140)	130 (126 to 150)	0.5383
DBP(mmHg) ^c^	80 (70 to 80)	80 (71 to 80)	0.2940
Smoking ^b^	15 (22.4)	6 (12.0)	0.1475
PAD ^b^	6 (9.0)	1 (2.0)	0.2361
Previous MI ^b^	38 (56.7)	24 (48.0)	0.4541
Hypertension ^b^	53 (79.1)	45 (90.0)	0.1139
Atrial fibrillation ^b^	4 (6.0)	16 (32.0)	0.00021 *
Paroxysmal atrial fibrillation ^b^	7 (10.4)	4 (8.0)	0.7563
CCS ^b^	24 (35.8)	14 (28.0)	0.3715
COPD ^b^	0 (0.0)	10 (20.0)	0.0001 *
Previous stroke ^b^	6 (9.0)	4 (8.0)	1.000
Obesity (BMI ≥ 30 kg/m^2^) ^b^	35 (52.2)	37 (74.0)	0.0167 *
Coronary angiography ^b^			0.350
Without lesions	4 (6.0)	1 (2.0)	
1-vessel CAD	17 (25.4)	14 (28.0)	
2-vessel CAD	21 (31.3)	22 (44.0)	
3-vessel CAD	25 (37.3)	13 (26.0)	
T2DM severity ^b^			0.1149
Controlled (HbA1c < 7%)	19 (28.4)	8 (16.0)	
Moderately controlled (7 ≤ HbA1c < 8%)	14 (20.9)	18 (36.0)	
Uncontrolled (HbA1c ≥ 8%)	34 (50.7)	24 (48.0)	

Data are presented as ^a^ arithmetic mean (standard deviation) or ^b^ absolute frequencies (relative frequencies, %) or ^c^ median [25th percentile; 75th percentile]; *p*-values were obtained by Student’s *t*-test with equal variances, Welch’s *t*-test, Chi-square test or Fisher’s Exact test; * significant results (*p* < 0.05); SBP—systolic blood pressure, DBP—diastolic blood pressure, PAD—peripheral artery disease, MI—myocardial infarction, CCS—chronic coronary syndrome, COPD—chronic obstructive pulmonary disease, CAD—coronary artery disease, T2DM—type 2 diabetes mellitus, HbA1c—glycated hemoglobin.

**Table 2:** Paraclinical features and treatments stratified by HF severity.

	Mild HF (n = 67)	Advanced HF (n = 50)	p-Value
Medication ^a^			
Metformin	56 (83.6)	34 (68.0)	0.04780 *
GLP-1 receptor agonist	2 (3.0)	6 (12.0)	0.07169
SGLT2 inhibitor	12 (17.9)	11 (22.0)	0.58190
Insulin	32 (47.8)	33 (66.0)	0.04952 *
Laboratory parameters			
Hemoglobin ^b^, g/dL	13.65 (1.62)	12.87 (1.94)	0.01934 *
Creatinine ^c^, mg/dL	0.87 [0.72, 1.12]	1.09 [0.84, 1.46]	0.00381 *
eGFR ^c^, mL/min/1.73 m^2^ by CKD-EPI	83.0 [59.0, 101.0]	69.5 [46.25, 86.75]	0.01226 *
Urea ^c^, mg/dL	38.95 [30.32, 54.50]	53.59 [38.49, 73.60]	0.00389 *
Uric acid ^b^, mg/dL	6.29 (1.58)	7.25 (2.12)	0.00882 *
HDL cholesterol ^b^, mg/dL	8.47 (2.03)	8.30 (1.59)	0.29790
Total cholesterol ^c^, mg/dL	164.0 [136.0, 204.0]	153.5 [122.0, 194.3]	0.17010
LDL cholesterol ^c^, mg/dL	96.0 [69.10, 123.30]	84.5 [61.85, 113.45]	0.44050
HbA1c ^b^, %	8.47 (2.03)	8.30 (1.59)	0.62960
Glycemia ^b^, mg/dL	178.90 (72.26)	191.24 (80.82)	0.42620

Data are presented as ^a^ absolute frequencies (relative frequencies, %) or ^b^ arithmetic mean (standard deviation) or ^c^ median [25th percentile; 75th percentile]; *p*-values were obtained by Student’s *t*-test with equal variances, Welch’s *t*-test, Chi-square test or Fisher’s Exact test; * significant results (*p* < 0.05); GLP-1—Glucagon-like peptide-1, SGLT 2 inhibitors—sodium–glucose co-transporter-2 inhibitors, eGFR = estimated glomerular filtration rate; CKD-EPI = Chronic Kidney Disease Epidemiology Collaboration, HDL—high-density lipoprotein, HBA1c—glycated hemoglobin.

**Table 3:** Distributions of Copeptin, Galectin-3, FGF21, NT-proBNP and echocardiographic parameters in patients with mild and advanced heart failure.

Variables	Mild HF (n = 67)	Advanced HF (n = 50)	*p*-Value
Copeptin ^a^, pg/mL	215.11 [155.83, 528.82]	223.30 [153.85, 524.63]	0.7745
FGF21 ^a^, ng/mL	322.90 [178.70, 458.60]	580.30 [329.90, 865.03]	0.00027 *
Gal- 3 ^b^, ng/mL	12.06 (5.49)	12.92 (4.16)	0.3327
NT-proBNP ^a^, pg/mL	1012.0 [327.0, 1704.5]	2515.5 [1178.5, 3843.75]	0.00003 *
LVEF ^b^, %	54.29 (6.08)	52.58 (6.84)	0.1576
LV EDV ^b^, mL	113.83 (33.03)	124.81 (39.51)	0.1047
LV ESV ^b^, mL	52.87 (20.03)	60.36 (23.88)	0.0680
LVDd ^b^, mm	50.67 (4.39)	50.42 (5.84)	0.7989
LVSd ^b^, mm	29.13 (5.46)	30.46 (7.32)	0.2846
E/e ‘ mean ratio ^b^	11.06 (2.77)	12.16 (4.01)	0.0997
Lad ^b^, mm	41.53 (5.67)	44.62 (5.52)	0.0039 *
LAV ^b^, mL	63.41 (17.81)	79.44 (26.10)	0.0003 *
LAVi ^a^, mL/m^2^	32.06 [26.92, 37.89]	35.31 [30.74, 43.21]	0.0126 *
LAS ^b^, cm^2^	22.95 (5.85)	26.18 (5.62)	0.0033 *
PWT ^b^, mm	11.97 (1.17)	12.14 (1.31)	0.4722
IVST ^b^, mm	12.36 (1.46)	12.26 (1.37)	0.7127
RWT ^b^	0.47 (0.05)	0.48 (0.08)	0.2992
EDT ^b^, msec	221.39 (42.76)	211.68 (38.36)	0.2071
TR velocity ^b^	2.45 (0.46)	2.76 (0.57)	0.0012 *
RV-RA gradient ^b^,mmHg	26.57 (8.41)	33.19 (11.79)	0.0011 *
PASP ^b^, mm HG	33.14 (9.10)	42.28 (14.77)	0.00023 *
LV GLS ^b^, %	13.69 (2.46)	13.08 (2.64)	0.2028

Data were described by ^a^ median (IQR) or ^b^ arithmetic mean (standard deviation); Gal-3—galectin 3, FGF21—fibroblast growth factor 21, LVEF—left ventricle ejection fraction, LVEDV—left ventricular end-diastolic volume, LVESV—left ventricular end-systolic volume, LAd—left atrium diameter, IVST—interventricular septum thickness, PWT—posterior wall thickness, LAV—left atrium volume, LAVi—left atrium volume index, LAS—left atrium surface, LVDd—left ventricular end-diastolic diameter, LVSd—left ventricular end-systolic diameter, EDT—E wave deceleration time, TR velocity—tricuspid regurgitation peak velocity, RV-RA—right ventricle–right atrium, PASP—pulmonary artery systolic pressure, LV GLS—left ventricle global longitudinal strain, * significant result: *p* < 0.05.

**Table 4:** Matrix of Spearman’s correlations between Copeptin, Galectin-3, FGF21, NT-proBNP and echocardiographic parameters stratified by severity of HF.

Groups /Variables	FGF21	NT-proBNP	Galectin-3	Copeptin
Advanced HF Group				
FGF21, ng/mL				
NTproBNP, pg/mL	−0.05 (0.7208)			
Gal-3, ng/mL	0.05 (0.7268)	−0.14 (0.3339)		
Copeptin, pg/mL	0.01 (0.9588)	−0.16 (0.9588)	−0.14 (0.3247)	
Age, years	−0.27 (0.0628)	0.31 (0.0273 *)	0.002 (0.9860)	0.004 (0.9781)
BMI, kg/m^2^	0.01 (0.9369)	−0.32 (0.0232 *)	0.18 (0.2096)	0.001 (0.9947)
SBP, mmHg	−0.10 (0.4840)	0.10 (0.5099)	0.20 (0.1603)	0.12 (0.3888)
DBP, mmHg	−0.06 (0.6971)	0.04 (0.7824)	−0.02 (0.9159)	0.10 (0.4800)
HbA1C, %	0.06 (0.7027)	−0.11 (0.4283)	0.07 (0.6054)	−0.13 (0.3588)
Hemoglobin, g/dL	−0.07 (0.6402)	−0.28 (0.0514)	0.002 (0.9884)	−0.19 (0.1928)
eGFR, mL/min/1.73 m^2^ by CKD-EPI	0.35 (0.0125 *)	−0.26 (0.0701)	−0.04 (0.760)	−0.007 (0.9592)
Urea, mg/dL	−0.16 (0.2581)	0.29 (0.0401 *)	0.18 (0.2194)	−0.14 (0.3446)
Uric acid, mg/dL	−0.19 (0.1779)	0.30 (0.0338 *)	0.20 (0.1674)	−0.17 (0.2371)
HDL cholesterol, mg/dL	−0.29 (0.0386 *)	0.08 (0.5583)	0.12 (0.3941)	−0.20 (0.1643)
Total cholesterol, mg/dL	0.22 (0.1196)	−0.32 (0.0247 *)	0.03 (0.8206)	0.04 (0.7751)
LDL cholesterol, mg/dL	0.21 (0.1413)	−0.29 (0.0379 *)	0.04 (0.7980)	0.09 (0.5170)
Triglycerides/dL	0.28 (0.0465 *)	−0.07 (0.6271)	−0.13 (0.3553)	0.02 (0.8660)
E/e’ ratio	−0.21 (0.1522)	−0.01 (0.9406)	0.09 (0.5542)	0.19 (0.1892)
LAVi, mL/m^2^	−0.15 (0.2891)	0.10 (0.4762)	0.08 (0.5780)	−0.03 (0.8415)
LAS, cm^2^	−0.23 (0.1141)	0.17 (0.2403)	0.02 (0.9144)	−0.08 (0.5954)
TR velocity, mmHg	−0.27 (0.0604)	0.38 (0.0068 *)	−0.18 (0.2231)	−0.10 (0.4696)
RV-RA gradient, mm Hg	−0.30 (0.0358 *)	0.31 (0.0301 *)	−0.17 (0.2329)	−0.09 (0.5015)
PASP, mmHg	−0.21 (0.1356)	0.39 (0.0053 *)	−0.12 (0.4058)	−0.14 (0.3399)
Mild HF Group				
FGF-21, ng/mL				
NTproBNP, pg/mL	−0.37 (0.0022 *)			
Gal-3, ng/mL	0.05 (0.6703)	−0.17 (0.1752)		
Copeptin, pg/mL	−0.02 (0.8829)	0.03 (0.7949)	−0.32 (0.0078 *)	
Age, years	−0.15 (0.2351)	0.11 (0.3965)	0.02 (0.8498)	0.22 (0.06725)
BMI, kg/m^2^	−0.04 (0.770)	−0.05 (0.6994)	0.46 (0.0001 *)	−0.25 (0.0417 *)
SBP, mmHg	−0.03 (0.7810)	0.14 (0.2508)	0.04 (0.7233)	−0.10 (0.4035)
DBP, mmHg	−0.06 (0.6085)	0.21 (0.0910)	−0.005 (0.9668)	0.008 (0.9477)
HbA1C, %	−0.09 (0.4530)	0.07 (0.5839)	−0.18 (0.1357)	−0.08 (0.4977)
Hemoglobin	0.06 (0.5888)	−0.08 (0.5294)	−0.29 (0.0154 *)	−0.15 (0.2304)
eGFR, mL/min/1.73 m^2^ by CKD-EPI	0.07 (0.5615)	−0.14 (0.2522)	−0.18 (0.1495)	−0.001 (0.9918)
Urea, mg/dL	0.17 (0.1569)	0.007 (0.9522)	0.06 (0.6266)	0.07 (0.5560)
Uric acid, mg/dL	−0.01 (0.9672)	0.15 (0.2388)	−0.04 (0.7275)	0.13 (0.3061)
HDL cholesterol/dL	0.01 (0.9321)	−0.21 (0.0936)	−0.19 (0.1299)	0.27 (0.0244 *)
Total cholesterol/dL	0.12 (0.3397)	−0.10 (0.4402)	−0.07 (0.5515)	0.12 (0.3459)
LDL cholesterol, mg/dL	0.05 (0.6688)	−0.03 (0.8322)	−0.06 (0.6107)	0.05 (0.6975)
Triglycerides, mg/dL	0.15 (0.2229)	−0.11 (0.3785)	0.06 (0.6341)	0.06 (0.6166)
E/e’ ratio	−0.29 (0.0182 *)	0.09 (0.4618)	0.10 (0.4289)	−0.08 (0.4973)
LAVi, mL/m^2^	−0.13 (0.3071)	0.05 (0.7004)	−0.05 (0.7148)	−0.11 (0.3625)
LAS, cm^2^	−0.15 (0.2358)	0.23 (0.0573)	0.13 (0.3045)	−0.24 (0.0523)
TR velocity, m/s	−0.24 (0.0470 *)	0.15 (0.2153)	0.08 (0.5289)	0.09 (0.4826)
RV-RA gradient, mmHg	−0.24 (0.0472 *)	0.12 (0.3256)	0.14 (0.2762)	0.03 (0.8407)
PAPS, mm HG	−0.23 (0.06014)	0.16 (0.2009)	0.12 (0.3217)	0.01 (0.9214)

Gal-3—galectin 3; FGF21—fibroblast growth factor 21; BMI—body mass index; SBP—systolic blood pressure; DBP—diastolic blood pressure; eGFR—estimated glomerular filtration rate; CKD-EPI—Chronic Kidney Disease Epidemiology Collaboration; HDL—high-density lipoprotein; LDL—low-density lipoprotein; HBA1c—glycated hemoglobin; LAV—left atrium volume; LAVi—left atrium volume index; LAS—left atrium surface; TR velocity—tricuspid regurgitation peak velocity; RV-RA—right ventricle–right atrium; PASP—pulmonary artery systolic pressure,* significant result: *p* < 0.05.

**Table 5:** Associations between FGF21 and NT-proBNP and odds of advanced HF in all tested models.

FGF21 (ng/mL)	OR [95% CI]	*p*-Value	NT-proBNP (pg/mL)	OR [95% CI]	*p*-Value
Model 1			Model 1		
<377.50	Reference		<2379	Reference	
≥377.50	4.46 [2.07, 10.94]	0.00019 *	≥2379	7.99 [3.29, 21.24]	0.00001 *
Model 2			Model 2		
<377.50	Reference		<2379	Reference	
≥377.50	5.06 [2.27, 12.03]	0.00012 *	≥2379	10.35 [3.92, 30.89]	0.000008 *
Model 3			Model 3		
<377.50	Reference		<2379	Reference	
≥377.50	8.19 [3.14, 24.03]	0.00004 *	≥2379	17.01 [5.11, 70.27]	0.00002 *

Model 1: univariable logistic binomial model. Model 2: multivariable logistic binomial model including the biomarker and age and sex as covariates. Model 3: multivariable logistic binomial model including the studied biomarker and age, sex, HT, obesity, smoking, atrial fibrillation and triglycerides ≥150 as covariates. OR = Odds Ratio; 95% CI = 95% confidence interval for OR; * significant result: *p* < 0.05.

## Data Availability

The raw data involved in this study can be obtained upon reasonable request addressed to Lucia M. Procopciuc (luciamariaprocopciuc@yahoo.com).
